# Screening of Crude Drugs Used in Japanese Kampo Formulas for Autophagy-Mediated Cell Survival of the Human Hepatocellular Carcinoma Cell Line

**DOI:** 10.3390/medicines6020063

**Published:** 2019-06-03

**Authors:** Shinya Okubo, Hisa Komori, Asuka Kuwahara, Tomoe Ohta, Yukihiro Shoyama, Takuhiro Uto

**Affiliations:** 1 Department of Pharmacognosy, Graduate School of Pharmaceutical Sciences, Nagasaki International University, Nagasaki 859-3298, Japan; shinyaokubo6252@yahoo.co.jp; 2 Department of Pharmacognosy, Faculty of Pharmaceutical Sciences, Nagasaki International University, Nagasaki 859-3298, Japan; tu19780217@gmail.com (H.K.); usa6050kuwa@gmail.com (A.K.); ohta@niu.ac.jp (T.O.); shoyama@niu.ac.jp (Y.S.)

**Keywords:** autophagic activity, screening, crude drugs, Kampo medicines, cell viability, LC3-II, p62, HepG2

## Abstract

**Background:** Autophagy is a catabolic process through which dysfunctional proteins and organelles are degraded, and that is associated with the proliferation of cancer cells. The aim of this study was to screen approximately 130 kinds of crude drugs used in Japanese Kampo formulas to identify crude drugs that would regulate the proliferation through autophagy of human hepatocellular carcinoma HepG2 cells. **Methods:** Extracts of each crude drug were prepared using methanol. Protein levels were determined using Western blotting. Cell viability was measured by MTT assay. **Results:** Among the 130 crude extracts, 24 of them increased LC3-II expression. Among these, Goboshi (burdock fruit), Soboku (sappan wood), Mokko (saussurea root), Rengyo (forsythia fruit), and Hikai (dioscorea) notably suppressed the proliferation of HepG2 cells and increased p62 expression levels, which suggested that these five extracts downregulate the autophagic activity resulting in the accumulation of p62. On the other hand, Hishinomi (water chestnut), Biwayo (loquat leaf), and Binroji (areca) induced cell growth and decreased or were uninvolved with p62 expression levels, which implied that these three extracts might induce autophagy modulators for cell growth. **Conclusions:** The results suggest that the compounds contained in the crude drugs selected for this study could control cell viability by regulating autophagic activity in HepG2 cells. The isolation and identification of the active compounds in these drugs might lead to the development of agents for autophagy research and cancer chemoprevention.

## 1. Introduction

Macroautophagy (hereafter, autophagy) is an evolutionarily conserved and self-consumption process that degrades cellular organelles and proteins and maintains cellular biosynthesis during nutrient deprivation or metabolic stress [1,2,3,4]. Accumulating evidence has indicated the importance of autophagy in various human diseases [2,3,4]. The steps in autophagy are initiation, phagophore elongation, autophagosome maturation, autophagosome fusion with the lysosome, and proteolytic degradation of the contents [1,2,3,4]. The products of degradation are recycled back into the cytosol and are reused to enhance cell survival during nutrient deprivation. In response to starvation, autophagy provides a nutrient source, promoting cell survival; however, autophagy is also induced by a broad range of other stressors and can degrade protein aggregates, oxidized lipids, and damaged organelles [1,2,3,4,5]. 

The entire autophagy process is complex and involves many critical proteins. Among these, LC3 and SQSTM1/p62 (p62) are responsible for autophagy’s membrane remodeling and trafficking events [6,7,8,9,10,11,12]. LC3 is an autophagosome membrane-bound protein first found in mammals [6]. Although there are several proteins that bind to the phagophore and autophagosome membranes, LC3 is widely used as a standard marker for these membranes because its binding is particularly stable [6]. After LC3 is synthesized as proLC3, it is immediately cleaved by cysteine protease to form LC3-I. Covalent binding of phosphatidylethanolamine to LC3-I results in LC3-II, which localizes to the phagophore and autophagosome membranes. The amount of LC3-II is proportional to the number of autophagosomes present within the cell [6,7]. p62 is a multifunctional signaling molecule involved in a variety of cellular pathways [8,9,10,11,12] that contains a ubiquitin-associated (UBA) domain for binding to ubiquitinated proteins. The presence of the UBA domain enables p62 to serve as an adaptor for selective autophagy of ubiquitinated substrates [8]. In addition, p62 itself can translocate not only to the phagophore membrane but also to the autophagosome formation site, even doing so independently of LC3 binding [12]. Therefore, p62 is a critical autophagic substrate and is widely used as an indicator of autophagic degradation [13].

In contrast to normal cells in tissues, tumors often locate within an environment deprived of nutrients, growth factors, and oxygen as a result of insufficient or abnormal vascularization. Thus, autophagy is important for supporting tumor growth [14,15,16,17]. In cancers, both the upregulation and downregulation of autophagic activity have been observed, which suggests its dual oncogenic and tumor-suppressing properties during malignant transformation [16,17]. Identification of the autophagy modulators is essential for the development of new therapeutic agents; however, no therapies are available that specifically focus on autophagy modulation. Although drugs such as chloroquine, hydroxychloroquine, and rapamycin act as autophagy modulators, they were not originally developed for this purpose. Thus, autophagy could represent a new and promising pharmacologic target in the development of future drugs and for therapeutic applications against human diseases, including cancer.

Recent studies have shown that natural products have beneficial effects on cancer therapy through autophagic activity [18,19,20,21,22]. Most of this research has reported that natural products induce autophagic activity in cancer cells. Berberine (BBR), an isoquinoline alkaloid isolated from *Coptis chinensis*, induces autophagic cell death by inhibiting the mammalian target of rapamycin complex 1 (mTORC1) through AMP-activated protein kinase (AMPK) activation in human hepatocellular carcinoma HepG2 cells [18]. BBR was also reported to induce autophagic cell death by enhancing glucose-regulated protein 78 (GRP78) levels in HepG2 cells and human colon carcinoma-116 and DLD1 cells [19]. Zanthoxylum fruit extracts from the Japanese pepper plant *Zanthoxylum piperitum* enhances autophagic cell death through the phosphorylation of c-Jun N-terminal kinase (JNK) in DLD1 cells [20]. On the other hand, it has recently been indicated that natural products have beneficial effects on cancer therapy by also downregulating autophagic activity. RA-XII, a natural cyclopeptide isolated from *Rubia yunnanensis*, suppresses protective autophagy to enhance apoptosis through AMPK/mTOR/70 kDa ribosomal protein S6 kinase (P70S6K) pathways in HepG2 cells [21]. Moreover, phyllanthusmins, which are isolated from various *Phyllanthus* species, inhibit late-stage autophagy by reducing lysosomal acidification, similar to the effects of bafilomycin A1 as a late-stage inhibitor of autophagy, and followed by apoptotic cell death in high-grade serous ovarian cancer (OVCAR3 and OVCAR8) cells [22]. These demonstrate the importance of identifying the modulators from natural products that both induce and inhibit autophagic activity to create new cancer therapy strategies.

Traditional Japanese Kampo medicine has been widely used in clinical practice in Japan. Although it was originally based on traditional Chinese medicine, Kampo medicine has developed more unique methods by which to diagnose and treat diseases. The Kampo formula is a combination of several crude drugs, most of which are derived from natural plants, but some of which are derived from animals and minerals. Parts of the crude drugs used in the Kampo formulas are also used in health foods and supplements. Kampo formulas have traditionally been prescribed for a number of health conditions, including chronic hepatitis, bronchial asthma, allergic rhinitis, anemia, and gastric cancer, and gradually reemerged in Japan as alternatives to Western medicine [23,24,25,26,27,28]. In a recent survey, approximately 90% of Japanese doctors prescribe Kampo formulas in their daily medical practice [29]. Kampo medicine enables physicians to deal with difficult-to-treat conditions by Western medicine alone. Moreover, each patient can grasp their own systemic state and improve their lifestyle. To extend healthy life expectancy, Kampo medicine plays an important role in health care in Japan [29]. Although several of the biological activities of the crude drugs used in the Kampo formulas were evaluated [30,31,32,33], there have been few reports that have investigated their effects on autophagic activity. In this study, we screened approximately 130 kinds of crude drugs used in the Kampo formulas in the comprehensive search for crude drugs exhibiting autophagy control in HepG2 cells. In addition, the effects of the selected crude extracts on cell viability were investigated to elucidate the relationship between modulation of autophagic activity and cancer cell viability.

## 2. Materials and Methods

### 2.1. Preparation of Crude Drug Extracts

All crude drugs were purchased from Yamaguchi Kampo Pharmacy (Nagasaki, Japan). Five grams were extracted from each crude drug overnight at room temperature using MeOH. The supernatant was evaporated under nitrogen gas to obtain the crude extract. The crude extracts were dissolved in dimethyl sulfoxide (DMSO) as a stock solution at the concentration of 100 mg/mL and stored at −20 °C.

### 2.2. Reagents

BBR chloride was purchased from Wako Pure Chemical Industries (Osaka, Japan). Antibodies against LC3B and SQSTM1/p62 were obtained from Cell Signaling Technology (Beverly, MA, USA). The antibody against β-actin and RIPA lysis buffer were purchased from Santa Cruz Biotechnology (Santa Cruz, CA, USA). Fetal bovine serum (FBS) was purchased from GIBCO (Gaithersburg, MD, USA). All other chemicals were obtained from Wako Pure Chemical Industries.

### 2.3. Cell Culture and Treatment

HepG2 cells were obtained from the RIKEN BioResource Center Cell Bank (Ibaraki, Japan). The cells were grown in Dulbecco’s Modified Eagle’s Medium supplemented with 10% FBS and 1% penicillin-streptomycin-L-glutamine and incubated at 37 °C with 5% CO_2_ under fully humidified conditions. During the cell treatments, the concentration of DMSO in the cell culture medium did not exceed 0.2% (*v*/*v*), and the controls were always treated with the same amount of DMSO as was used in the corresponding experiments.

### 2.4. Western Blot Analysis

Cells (1 × 10^6^ cells/dish) were plated on 6 cm dishes. After replacing with fresh medium, the cells were treated with each crude extract or BBR for various time periods, after which they were harvested and lysed in RIPA lysis buffer containing protease and phosphatase inhibitors. After centrifuging for 15 min at 12,000× *g* and 4 °C, the protein content of the samples was determined using a dye-binding protein assay kit according to the manufacturer’s instructions (Bio-Rad, Richmond, CA, USA). Equal amounts of lysate protein were subjected to SDS-PAGE. The proteins were electrotransferred to PVDF membranes and detected as previously described [30]. BBR was then used as a positive control [18,19]. The relative intensity of the indicated band was quantified using ImageJ software (1.50i; Java 1.6.0_24 (64-bit), National Institutes of Health, Bethesda, MD, USA), and the value was normalized to a corresponding loading control and expressed as the fold change in the control group.

### 2.5. Determination of Cell Viability

Cell viability was determined using a 3-(4,5-dimethylthiazol-2-yl)-2,5-diphenyltetrazolium bromide (MTT) assay and 0.9 × 10^4^ cells/well were cultured on 96-well plates. After replacing the original medium with fresh medium, the cells were treated with various concentrations of each crude extract for 24 h. At the end of treatment, 10 μL of 5 mg/mL MTT solution were added to each well, and the cells were incubated for another 4 h. The precipitated MTT-formazan was dissolved in 100 μL of 0.04 N HCl-isopropanol, and the amount of formazan was measured at 595 nm using an iMark microplate reader (Bio-Rad, Tokyo, Japan). Cell viability was expressed as a percentage of the control culture.

### 2.6. Statistical Analyses

All data were derived from at least three independent treatment repetitions. The results are expressed as the mean ± SD under each condition. Differences among the groups were analyzed using Student’s *t*-test. *p* < 0.005 was considered statistically significant.

## 3. Results

### 3.1. Screening the Crude Drugs for LC3-II Expression

**Figure 1:**
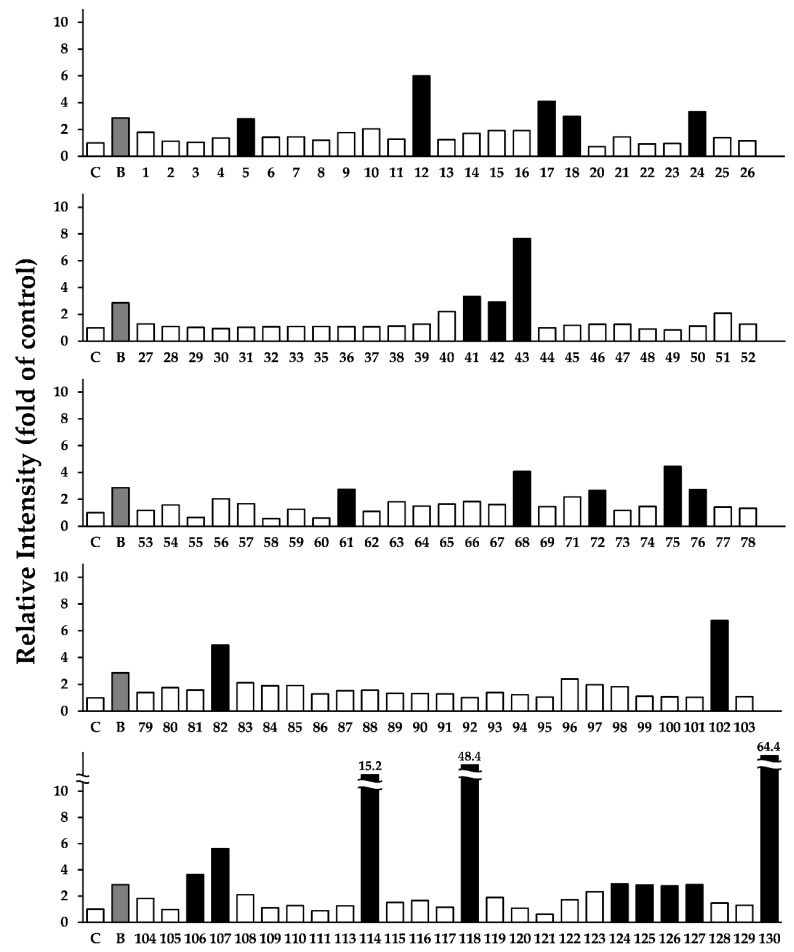
Relative intensity of LC3-II protein levels by the approximately 130 kinds of crude extracts. The noticeable increase is shown in the black column. HepG2 cells were treated with 20 µg/mL of each crude extract for 24 h, and the LC3-II expression levels were determined using Western blotting. The data shown are representative of three independent treatments with similar results. Notes: C, control; B, positive control (BBR 50 μM); 1–130, crude drug number (see Table S1, Supplementary Materials).

Table S1 (Supplementary Materials) shows the crude drugs used in this study. The listed crude drugs cover not only those in Japanese Pharmacopoeia but also those commonly used in medical therapy in Japan. We first investigated the effects of MeOH extracts prepared from the crude drugs on the expression of LC3-II in HepG2 cells. The cells were treated with the approximately 130 kinds of crude extracts at 20 μg/mL for 24 h. LC3-II protein levels were examined using Western blotting, and the band intensities were quantified and expressed as the fold changes in the control. BBR, which induces autophagy and increases LC3-II expression in HepG2 cells, was used as a positive control [18,19]. As shown in Figure 1 and Figure S1, among the 130 crude extracts, 24 crude extracts, which are shown in black columns, increased LC3-II expression, which suggests that they might exhibit autophagic activity.

### 3.2. Effects of the Selected 24 Crude Drugs on Cell Viability

**Figure 2:**
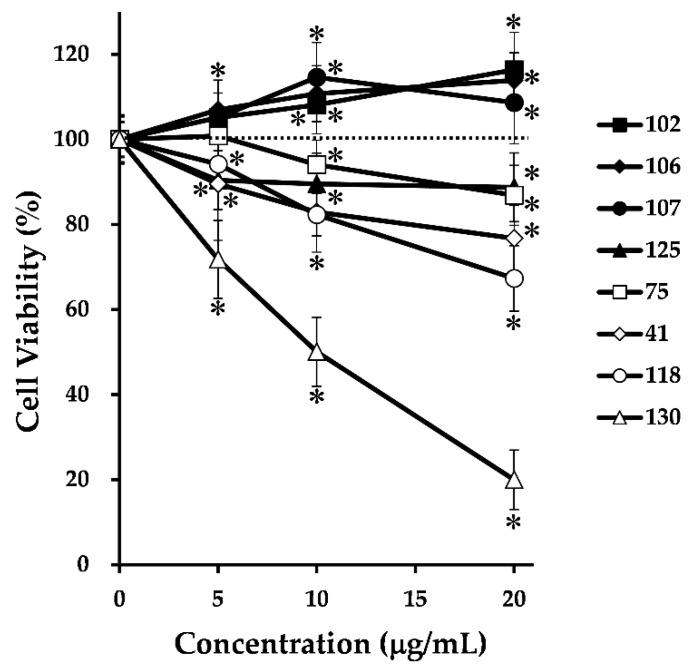
Effects of eight crude extracts on the proliferation of HepG2 cells. Cells were treated with each crude extract at various concentrations for 24 h, and cell viabilities were determined using MTT assay. The data represent the mean ± SD of three individual treatments using the same concentrations. ∗ *p* < 0.005 compared with the control group.

It has been suggested that autophagic activity is intimately related to the proliferation of cancer cells [14,15,16,17,18,19,20,21,22]; therefore, we next investigated the effects of the selected 24 crude drugs on the proliferation of HepG2 cells. The cells were treated with 5, 10, and 20 μg/mL of each of the 24 crude extracts for 24 h, and cell viability was measured using MTT assay. Table 1 indicates the percentage of cell viability after treatment with 5, 10, and 20 μg/mL of each extract. Among the 24 crude extracts, cell viability was significantly suppressed by >10% compared with that in the control using the following five kinds of crude extracts, namely, 41 (Goboshi; burdock fruit), 75 (Soboku; sappan wood), 118 (Mokko; saussurea root), 125 (Rengyo; forsythia fruit), and 130 (Hikai; dioscorea). On the other hand, three extracts, namely, 102 (Hishinomi; water chestnut), 106 (Biwayo; loquat leaf), and 107 (Binroji; areca), increased cell viability by >10% compared with that of the control. The remaining 16 crude extracts had no effect on cell viability. Figure 2 shows the cell viability of only eight crude extracts (five extracts that suppressed cell viability and three that increased cell viability). These results suggest that these eight crude drugs might influence cell viability by modulating autophagic activity. 

### 3.3. Effects of Eight Crude Drugs on P62 Expression

**Figure 3:**
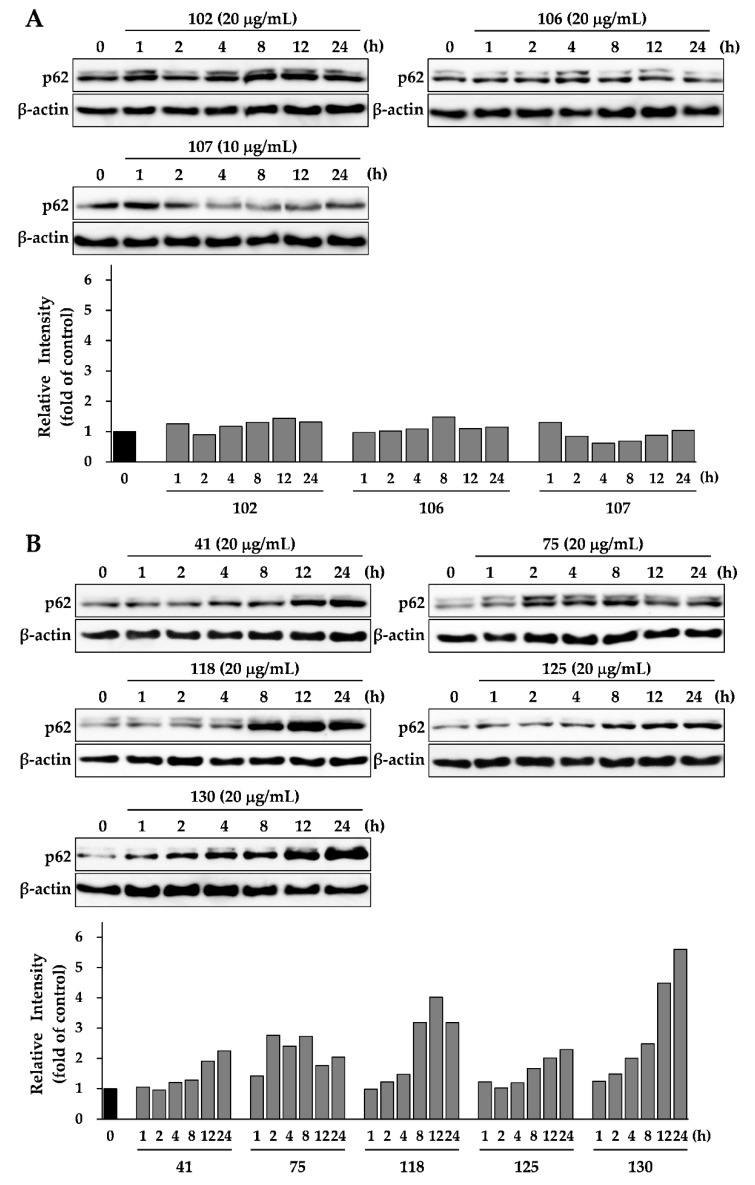
Effects of eight crude extracts on p62 expression in HepG2 cells. Cells were treated with each crude extract at the indicated concentrations and for different durations. The expressions levels of p62 and β-actin were determined by Western blotting. (**A**) The effect of crude extracts on cell viability. (**B**) The effect of crude extracts that suppressed cell viability. The data shown are representative of three independent treatments using the same parameters with similar results.

p62 is selectively incorporated into autophagosomes and is efficiently degraded by autophagy [8,9,10,11,12,13]; therefore, the p62 expression levels are inversely related to the autophagic activity. To identify whether the eight crude extracts in Figure 2 induce or inhibit autophagy, it was necessary to determine the autophagic flux assessed by monitoring p62 levels; therefore, we conducted a time-course experiment to determine the effect of the eight crude extracts on p62 expression levels. Figure 3A indicates the p62 expression levels regulated by the three crude extracts that increased cell viability. Crude drug numbers 102 and 106 had no effect on p62 expression levels during treatment. Crude drug number 107 decreased p62 expression level within a short time (approximately 4 h) after treatment and then p62 expression recovered to basal levels after 12 h. Previous studies have reported that the impairment of autophagy causes massive accumulation of p62 in HepG2 cells [13,21]. Conversely, when autophagy is activated, p62 rapidly degrades but then recovers to basal levels after several hours [34]. The results of these studies suggest that crude drug numbers 102, 106, and 107 did not block autophagic flux. Furthermore, the crude drug number 107 clearly induced autophagy. Figure 3B shows the p62 expression levels of treatment with five crude extracts that suppressed cell viability. All of these crude extracts increased p62 expression levels over time. In particular, crude drug number 130 strongly induced p62 expression levels by more than fourfold after treatments for 12 and 24 h. Crude drug number 118 also induced p62 expression by more than fourfold after treatment for 12 h. These results suggest that crude drug numbers 41, 75, 118, 125, and 130 deregulate the autophagic pathway by blocking autophagic flux, which results in p62 accumulation.

## 4. Discussion

Liver cancer is the second leading cause of cancer-related death and the sixth most diagnosed cancer worldwide [35]. Autophagy plays multiple roles in maintaining liver homeostasis. In the absence of Atg5 and Atg7, which are key autophagy genes involved in its initiation, nonfunctional proteins and organelles accumulate in liver cells [36]. It has been reported that conditional Atg7 knockout mice developed hepatomegaly and different metabolic liver disorders [37]; therefore, autophagy is important for suppressing tumorigenesis in the liver. Cancer cells use autophagy to ensure an alternative energy source for growth and survival in a stressful microenvironment with high hypoxia, scarce nutrients, and, very often, under stressful therapeutic conditions [14,15,16,17]. It has been reported that in advanced hepatocellular carcinomas, autophagy plays an oncogenic (pro-survival) role observed as an increased LC3-II expression that positively correlates with progression of malignancy and a poor prognosis [38]. However, one should be careful when coming to conclusions about the influence of LC3-II overexpression on autophagic activity. Hence, LC3-II overexpression cannot be solely used as a marker for increased autophagic activity because its increased accumulation might have resulted from autophagy inhibition at the post-lipidation stage.

In this study, we screened approximately 130 kinds of crude drugs used in Japanese Kampo formulas to identify those drugs that regulate HepG2 proliferation through autophagy. First, we screened 130 crude extracts to select those that increased LC3-II expression. Among them, 24 crude extracts did so, which suggests that these extracts might have autophagic activity. Among the 24 crude extracts that increased LC3-II expression, three induced cell growth but five suppressed cell viability. Interestingly, the remaining 16 crude extracts had no effect on cell viability despite LC3-II upregulation. These results suggest that the regulation of HepG2 proliferation is not indispensable for LC3-II induction. Because the results of screening for LC3-II expression cannot be directly applied to the selection of extracts that inhibit autophagy, we determined that autophagic flux could be assessed by monitoring p62 expression levels. Among the three crude extracts that increased cell viability, crude drug numbers 102 and 106 had no effect on p62 expression levels, and crude drug number 107 decreased p62 expression levels within a short duration after treatment, but then the levels finally recovered to their basal levels. It appears that crude drug number 107 induced cell growth by inducing autophagic activity, and we expect that this crude drug might contain compounds that are specific activators of autophagy. Although crude drug numbers 102 and 106 did not show obvious induction of autophagic activity, they showed that LC3-II expression can be increased without blocking autophagic flux. These results probably reflect the conclusion that crude drug numbers 102 and 106 induced cell growth by inducing autophagic activity, but further investigations are needed to confirm the findings. In fact, crude drug number 107 (Binroji; areca) has been reported to induce autophagy in several cell lines, such as leukemic Jurkat T cells [39] and oral carcinoma cells (OECM-1), Cal-27, and Scc-9 [40] by activating the AMPK/mTOR signaling pathway after accumulating reactive oxygen species [40]. However, the relationship between autophagy and crude drug numbers 102 (Hishinomi; water chestnut) or 106 (Biwayo; loquat leaf) has not been previously reported.

On the other hand, the five crude extracts that suppressed cell viability increased p62 expression levels, which suggests that these extracts downregulate the autophagic pathway by inhibiting autophagic flux, leading to p62 accumulation. Among these five extracts, crude drug numbers 41 (Goboshi; burdock fruit) and 125 (Rengyo; forsythia fruit) contain the same major compound, arctigenin (AG) of lignans. It has been reported that AG protects against drug-induced hepatitis by suppressing the immune system and regulating autophagy by inhibiting the IFN-γ/IL-6/Stat1 and IL-6/Bnip3 pathways in mice [41]. However, our results in this study indicate that crude drug numbers 75 (Soboku; sappan wood) and 118 (Mokko; saussurea root) reduced autophagic activity with p62 accumulation. The brazilin contained in crude drug number 75 was reported to induce autophagic cell death by disturbing calcium homeostasis in osteosarcoma MG-63 cells, thereby suppressing cell viability [42]. The costunolide contained in crude drug number 118 was reported to inhibit cell viability of multidrug-resistant human ovarian cancer OAW42-A cells by activating apoptotic and autophagic pathways via the decreased expression of B-cell lymphoma 2 (Bcl-2) [43]. This implies that the differences in the reactivity and signaling pathways depend on the cell type. Our results indicate, and those of previous reports have indicated, that these extracts contain compounds that modulate autophagic activity. In addition, our findings support the possibility that these crude extracts regulate autophagic activity to recover the medical conditions of patients, and provide new insights into pharmacological action underlying Japanese Kampo formulas in future investigations. We conclude that the crude drugs selected for this study could serve as sources of lead compounds in the development of agents for autophagy research and cancer therapy, and that, additional studies are needed to isolate and identify the active compounds in the drugs.

## Figures and Tables

**Table 1:** Effects of selected 24 crude extracts on the proliferation.

Drug No.	Japanese Name	English Name	Concentration (µg/mL)		
			5	10	20
5	Uzu	Aconite Root	100.4	100.3	103.3
12	Onji	Polygala Root	100.4	97.3	96.4
17	Kakko	Pogostemon Herb	96.6	100.2	100.5
18	Kakkon	Pueraria Root	101.6	107.4	109.5
24	Kikyo	Platycodon Root	103.6	106.9	106.8
41 ^(b)^	Goboshi	Burdock Fruit	89.5	82.9	76.7
42	Gomishi	Schisandra Fruit	105.5	107.3	106.2
43	Saiko	Bupleurum Root	100.8	100.3	96.1
61	Jashoshi	Cnidium Monnieri Fruit	96.1	96.1	92.6
68	Shoma	Cimicifuga Rhizome	100.2	104.3	106.3
72	Sentai	Cicada Slough	105.6	103.2	101.4
75 ^(b)^	Soboku	Sappan Wood	100.8	94.0	86.9
76	Soyo	Perilla Herb	102.7	103.8	105.9
82	Chimo	Anemarrhena Rhizome	99.4	100.8	96.9
102 ^(a)^	Hishinomi	Water Chestnut	105.1	108.1	116.3
106 ^(a)^	Biwayo	Loquat Leaf	106.9	110.7	113.9
107 ^(a)^	Binroji	Areca	105.1	114.6	108.7
114	Mao	Ephedra Herb	101.5	95.0	97.7
118 ^(b)^	Mokko	Saussurea Root	94.2	82.3	67.3
124	Ryokyo	Alpinia Officinarum Rhizome	101.4	103.2	109.5
125 ^(b)^	Rengyo	Forsythia Fruit	90.4	89.5	88.7
126	Renniku	Nelumbo Seed	103.5	105.6	103.2
127	Tanjin	Salvia Miltiorrhiza Root	98.2	99.8	95.3
130 ^(b)^	Hikai	Dioscorea	71.8	50.1	20.0

^(a)^ Extracts that increased cell viability by >10% compared with the control. ^(b)^ Extracts that suppressed cell viability by >10% compared with the control.

## Supplementary Materials

The following are available online at https://www.mdpi.com/2305-6320/6/2/63/s1, Table S1: List of crude drugs, Figure S1: The effect of crude extracts including 24 selected crude drugs on LC3-II protein.
