# Supplementary material for: Screening of Crude Drugs Used in Japanese Kampo Formulas for Autophagy-Mediated Cell Survival of the Human Hepatocellular Carcinoma Cell Line
Source: Medicines (Basel). 2019 Jun 3;6(2):63. doi: 10.3390/medicines6020063 (PMC6631990; doi:10.3390/medicines6020063)
Supplement: Supplementary file 1 [file medicines-06-00063-s001.pdf]

# Supplementary Materials: Screening of Crude Drugs Used in Japanese Kampo Formulas for Autophagy-Mediated Cell Survival of the Human Hepatocellular Carcinoma Cell Line

**Shinya Okubo, Hisa Komori, Asuka Kuwahara, Tomoe Ohta, Yukihiro Shoyama and Takuhiro Uto**

**Table S1.** List of crude drugs.

| Drug No. | Japanese Name | English Name | Scientific Name | Medicinal Part |
|---|---|---|---|---|
| 1 | Akyo | Donkey Glue | *Equus asinus* | glue |
| 2 | Ireisen | Clematis Root | *Clematis chinensis, C. mandshurica, C. hexapetala* | root with rhizome |
| 3 | Inchinko | Artemisia Capillaris Flower | *Artemisia capillaris* | capitulum |
| 4 | Uikyo | Fennel | *Foeniculum vulgare* | fruit |
| 5 | Uzu [a)] | Aconite Root | *Aconitum carmichaeli, A. japonicum* | tuberous root (mother root) |
| 6 | Uyaku | Lindera Root | *Lindera strychnifolia* | root |
| 7 | Engosaku | Corydalis Tuber | *Corydalis turtschaninovii* | tuber |
| 8 | Ogi | Astragalus Root | *Astragalus membranaceus, A. mongholicus* | root |
| 9 | Ogon | Scutellaria Root | *Scutellaria baicalensis* | root |
| 10 | Obaku | Phellodendron Bark | *Phellodendron amurense, P. chinense* | bark |
| 11 | Oren | Coptis Rhizome | *Coptis japonica, C. chinensis, C. deltoidea, C. teeta* | rhizome |
| 12 | Onji | Polygala Root | *Polygala tenuifolia* | root or root bark |
| 13 | Gaiyo | Artemisia Leaf | *Artemisia princeps, A. montana* | leaf and twig |
| 14 | Kashi | Myrobalan Fruit | *Terminalia chebula* | fruit |
| 15 | Kashu | Polygonum Root | *Polygonum multiflorum* | root |
| 16 | Gajutsu | Zedoary | *Curcuma zedoaria* | rhizome |
| 17 | Kakko | Pogostemon Herb | *Pogostemon cablin* | aerial part |
| 18 | Kakkon | Pueraria Root | *Pueraria lobata* | root |
| 19 | Kasseki | Aluminum Silicate Hydrate with Silicon Dioxide | — | — |
| 20 | Karokon | Trichosanthes Root | *Trichosanthes kirilowii, T. kirilowii* var. *japonica, T. bracteata* | root |

| | | | | |
|---|---|---|---|---|
| 21 | Karonin | Trichosanthes Seed | *Trichosanthes kirilowii, T. kirilowii* var. *japonica, T. bracteata* | seed |
| 22 | Kankyo [b)] | Processed Ginger | *Zingiber officinale* | rhizome |
| 23 | Kanzo | Glycyrrhiza | *Glycyrrhiza uralensis, G. glabra* | root and stolon |
| 24 | Kikyo | Platycodon Root | *Platycodon grandiflorum* | root |
| 25 | Kikuka | Chrysanthemum Flower | *Chrysanthemum morifolium, C. indicum* | capitulum |
| 26 | Kijitsu | Immature Orange | *Citrus aurantium* var. *daidai, C. aurantium, C. natsudaidai* | fruit |
| 27 | Kyokatsu | Notopterygium | *Notopterygium incisum, N. forbesii* | rhizome and root |
| 28 | Kyonin | Apricot Kernel | *Prunus armeniaca, P. armeniaca* var. *ansu, P. sibirica* | seed |
| 29 | Kinginka | Lonicera Flower | *Lonicera japonica* | flower bud |
| 30 | Kukoshi | Lycium Fruit | *Lycium chinense, L. barbarum* | fruit |
| 31 | Kujin | Sophora Root | *Sophora flavescens* | root |
| 32 | Keigai | Schizonepeta Spike | *Schizonepeta tenuifolia* | spike |
| 33 | Keihi | Cinnamon Bark | *Cinnamomum cassia* | bark |
| 34 | Kengoshi | Pharbitis Seed | *Pharbitis nil* | seed |
| 35 | Genjin | Scrophularia Root | *Scrophularia ningpoensis, S. buergeriana* | root |
| 36 | Koka | Safflower | *Carthamus tinctorius* | tubulous flower |
| 37 | Kobushi | Cyperus Rhizome | *Cyperus rotundus* | rhizome |
| 38 | Koboku | Magnolia Bark | *Magnolia obovata, M. officinalis, M. officinalis* var. *biloba* | bark |
| 39 | Goshitsu | Achyranthes Root | *Achyranthes fauriei, A. bidentata* | root |
| 40 | Goshuyu | Euodia Fruit | *Euodia ruticarpa, E. officinalis, E. bodinieri* | fruit |
| 41 | Goboshi | Burdock Fruit | *Arctium lappa* | fruit |
| 42 | Gomishi | Schisandra Fruit | *Schisandra chinensis* | fruit |
| 43 | Saiko | Bupleurum Root | *Bupleurum falcatum* | root |
| 44 | Saishin | Asiasarum Root | *Asiasarum sieboldii, A. heterotropoides* var. *mandshuricum* | root with rhizome |
| 45 | Sankirai | Smilax Rhizome | *Smilax glabra* | tuber |
| 46 | Sanzashi | Crataegus Fruit | *Crataegus cuneata, C. pinnatifida* var. *major* | pseudocarp |
| 47 | Sanshishi | Gardenia Fruit | *Gardenia jasminoides* | fruit |
| 48 | Sanshuyu | Cornus Fruit | *Cornus officinalis* | pulp of pseudocarp |
| 49 | Sansho | Japanese Zanthoxylum Peel | *Zanthoxylum piperitum* | pericarp |
| 50 | Sansonin | Jujube Seed | *Zizyphus jujuba* var. *spinosa* | seed |
| 51 | San'yaku | Dioscorea Rhizome | *Dioscorea japonica, D. batatas* | rhizome |
| 52 | Jio [c)] | Rehmannia Root | *Rehmannia glutinosa* var. *purpurea, R. glutinosa* | root |
| 53 | Jukujio [c)] | Rehmannia Root | *Rehmannia glutinosa* var. *purpurea, R. glutinosa* | root |

| | | | | |
|---|---|---|---|---|
| 54 | Shion | Aster Root | *Aster tataricus* | root and rhizome |
| 55 | Jikoppi | Lycium Bark | *Lycium chinense, L. barbarum* | root bark |
| 56 | Shikon | Lithospermum Root | *Lithospermum erythrorhizon* | root |
| 57 | Shisoshi | Perilla Fruit | *Perilla frutescens* var. *crispa* | fruit |
| 58 | Shitsurishi | Tribulus Fruit | *Tribulus terrestris* | fruit |
| 59 | Shitei | Persimmon Calyx | *Diospyros kaki* | calyx |
| 60 | Shakuyaku | Peony Root | *Paeonia lactiflora* | root |
| 61 | Jashoshi | Cnidium Monnieri Fruit | *Cnidium monnieri* | fruit |
| 62 | Shajin | Adenophora Root | *Adenophora tetraphylla, A. stricta, A. triphylla, A. hunanensis* | root |
| 63 | Shazenshi | Plantago Seed | *Plantago asiatica* | seed |
| 64 | Shazenso | Plantago Herb | *Plantago asiatica* | entire plant |
| 65 | Shukusha | Amomum Seed | *Amomum xanthioides* | seed mass |
| 66 | Shokyo [b)] | Ginger | *Zingiber officinale* | rhizome |
| 67 | Shobaku | Wheat | *Triticum aestivum* | fruit |
| 68 | Shoma | Cimicifuga Rhizome | *Cimicifuga simplex, C. dahurica, C. foetida, C. heracleifolia* | rhizome |
| 69 | Shin'i | Magnolia Flower | *Magnolia salicifolia, M. kobus, M. biondii, M. sprengeri, M. heptapeta* | flower bud |
| 70 | Sekko | Gypsum | — | — |
| 71 | Senkyu | Cnidium Rhizome | *Cnidium officinale* | rhizome |
| 72 | Sentai | Cicada Slough | *Cryptotympana atrata, Platylomia pieli, Oncotympana maculaticollis, Tanna chekiangensis, Graptopsaltria tienta, Lyristes pekinensis, L. atrofasciatus, Meimuna mongolica, Leptosemia sakaii, Platypleura kaempferi* or allied animals | cast-off shell |
| 73 | Sojutsu | Atractylodes Lancea Rhizome | *Atractylodes lancea, A. chinensis* | rhizome |
| 74 | Sohakuhi | Mulberry Bark | *Morus alba* | root bark |
| 75 | Soboku | Sappan Wood | *Caesalpinia sappan* | duramen |
| 76 | Soyo | Perilla Herb | *Perilla frutescens* var. *crispa* | leaf and tip of branch |
| 77 | Daio | Rhubarb | *Rheum palmatum, R. tanguticum, R. officinale, R. coreanum* | rhizome |
| 78 | Daihukuhi | Areca Pericarp | *Areca catechu, A. dicksonii* | pericarp |
| 79 | Taiso | Jujube | *Zizyphus jujuba* var. *inermis* | fruit |
| 80 | Takusha | Alisma Tuber | *Alisma orientale* | tuber |
| 81 | Chikujo | Bamboo Culm | *Bambusa textilis, B. pervariabilis, B. beecheyana, B. tuldoides, Phyllostachys nigra* var. *henonis, P. bambusoides* | inner layer of culm |

| | | | | |
|---|---|---|---|---|
| 82 | Chimo | Anemarrhena Rhizome | *Anemarrhena asphodeloides* | rhizome |
| 83 | Choji | Clove | *Syzygium aromaticum* | flower bud |
| 84 | Chotoko | Uncaria Hook | *Uncaria rhynchophylla, U. sinensis, U. macrophylla* | hook |
| 85 | Chorei | Polyporus Sclerotium | *Polyporus umbellatus* | sclerotium |
| 86 | Chimpi | Citrus Unshiu Peel | *Citrus unshiu, C. reticulata* | pericarp |
| 87 | Tennansho | Arisaema Tuber | *Arisaema heterophyllum, A. erubescens, A. amurense* | tuber |
| 88 | Temma | Gastrodia Tuber | *Gastrodia elata* | tuber |
| 89 | Temmondo | Asparagus Root | *Asparagus cochinchinensis* | root |
| 90 | Togashi | Benincasa Seed | *Benincasa cerifera, B. cerifera* forma *emarginata* | seed |
| 91 | Toki | Japanese Angelica Root | *Angelica acutiloba, A. acutiloba* var. *sugiyamae* | root |
| 92 | Todokukatsu | Angelica Pubescens Root | *Angelica pubescens, A. biserrata* | root |
| 93 | Tonin | Peach Kernel | *Prunus persica, P. persica* var. *davidiana* | seed |
| 94 | Dokukatsu | Aralia Rhizome | *Aralia cordata* | rhizome |
| 95 | Tochu | Eucommia Bark | *Eucommia ulmoides* | bark |
| 96 | Ninjin | Ginseng | *Panax ginseng* | root |
| 97 | Baimo | Fritillaria Bulb | *Fritillaria verticillata* var. *thunbergii* | bulb |
| 98 | Bakuga | Malt | *Hordeum vulgare* | caryopsis |
| 99 | Bakumondo | Ophiopogon Root | *Ophiopogon japonicus* | enlarged part of root |
| 100 | Hakka | Mentha Herb | *Mentha arvensis* var. *piperascens* | aerial part |
| 101 | Hange | Pinellia Tuber | *Pinellia ternata* | tuber |
| 102 | Hishinomi | Water Chestnut | *Trapa japonica, T. incisa,T. japonica* var. *rubeola* | fruit |
| 103 | Byakugo | Lilium Bulb | *Lilium lancifolium, L. brownii* var. *colchesteri, L. brownie, L. pumilum* | scaly leaf |
| 104 | Byakushi | Angelica Dahurica Root | *Angelica dahurica* | root |
| 105 | Byakujutsu | Atractylodes Rhizome | *Atractylodes japonica, A. macrocephala* | rhizome |
| 106 | Biwayo | Loquat Leaf | *Eriobotrya japonica* | leaf |
| 107 | Binroji | Areca | *Areca catechu* | seed |
| 108 | Bukuryo | Poria Sclerotium | *Wolfiporia cocos* | sclerotium |
| 109 | Boi | Sinomenium Stem and Rhizome | *Sinomenium acutum* | stem and rhizome |
| 110 | Bokon | Imperata Rhizome | *Imperata cylindrica* | rhizome |
| 111 | Bofu | Saposhnikovia Root and Rhizome | *Saposhnikovia divaricata* | root and rhizome |

| | | | | |
|---|---|---|---|---|
| 112 | Hobushi [a)] | Processed Aconite Root | *Aconitum carmichaeli, A. japonicum* | tuberous root (daughter root) |
| 113 | Botampi | Moutan Bark | *Paeonia suffruticosa* | root bark |
| 114 | Mao | Ephedra Herb | *Ephedra sinica, E. intermedia, E. equisetina* | aerial stem |
| 115 | Mashinin | Hemp Fruit | *Cannabis sativa* | fruit |
| 116 | Mankeishi | Shrub Chaste Tree Fruit | *Vitex rotundifolia, V. trifolia* | fruit |
| 117 | Mokutsu | Akebia Stem | *Akebia quinata, A. trifoliata* | stem |
| 118 | Mokko | Saussurea Root | *Saussurea lappa* | root |
| 119 | Yakuchi | Bitter Cardamon | *Alpinia oxyphylla* | fruit |
| 120 | Yakumoso | Leonurus Herb | *Leonurus japonicus, L. sibiricus* | aerial part |
| 121 | Yokuinin | Coix Seed | *Coix lachryma-jobi* var. *mayuen* | seed |
| 122 | Ryugan'niku | Longan Aril | *Euphoria longana* | aril |
| 123 | Ryutan | Japanese Gentian | *Gentiana scabra, G. manshurica, G. triflora* | root and rhizome |
| 124 | Ryokyo | Alpinia Officinarum Rhizome | *Alpinia officinarum* | rhizome |
| 125 | Rengyo | Forsythia Fruit | *Forsythia suspensa* | fruit |
| 126 | Renniku | Nelumbo Seed | *Nelumbo nucifera* | seed |
| 127 | Tanjin | Salvia Miltiorrhiza Root | *Salvia miltiorrhiza* | root |
| 128 | Hanshiren | Barbated Skullcup Herb | *Scutellaria barbata* | entire plant |
| 129 | Byakkajazetsuso | Oldenlandia diffusa | *Hedyotis diffusa* var. *longipe* | entire plant |
| 130 | Hikai | Dioscorea | *Dioscorea tokoro* | rhizome |

a,b,c) specific processing is different

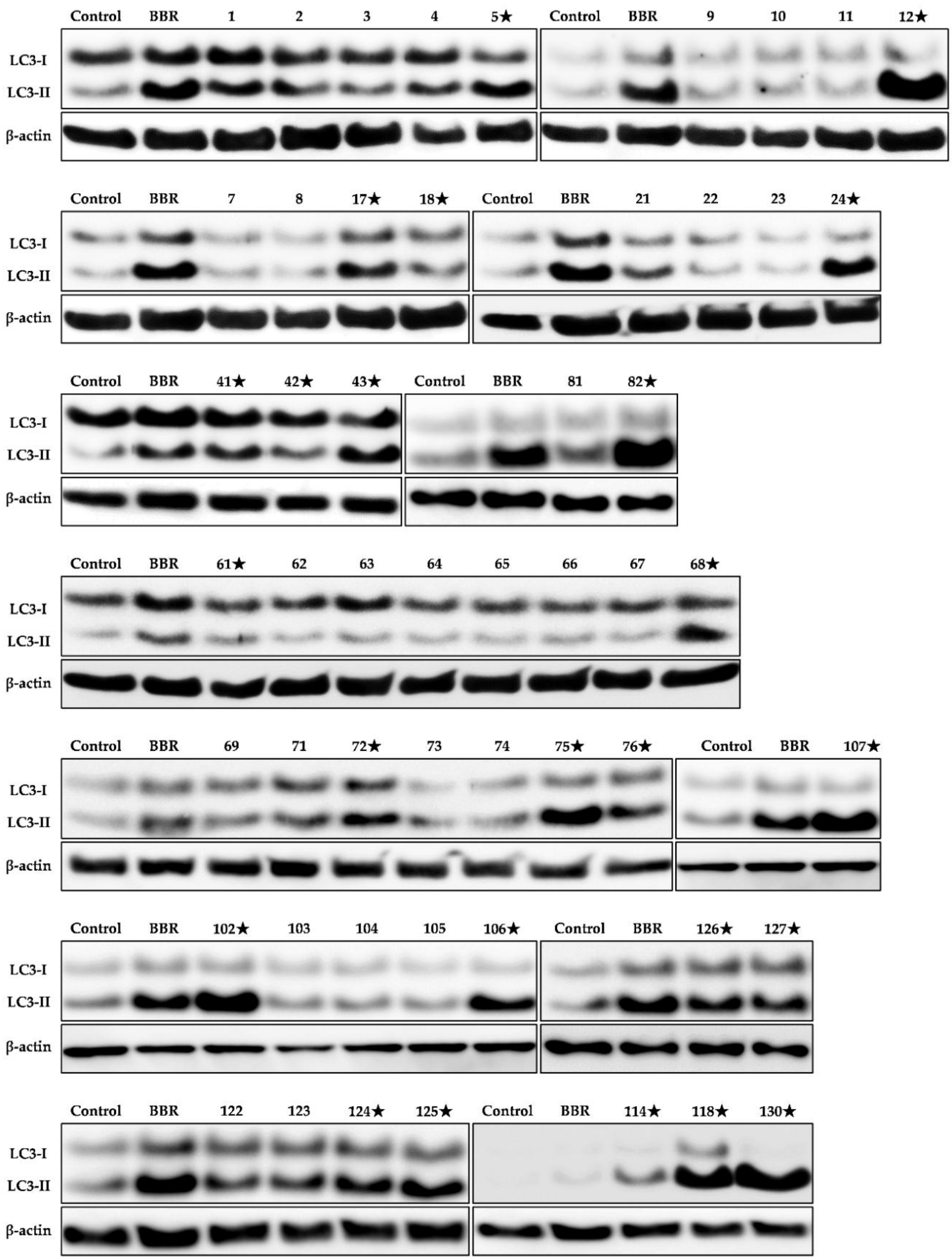


**Figure S1.** The effect of crude extracts including 24 selected crude drugs on LC3-II protein. The 24 selected crude drugs are shown in the black star (★). HepG2 cells were treated with 20 μg/mL of each crude extract for 24 h, and the LC3-II expression levels were determined using Western blotting. The data shown are representative of three independent treatments with similar results. Notes: BBR; positive control (BBR 50 μM), 1–130; crude drug number (see Table S1).
